# Disease modifying treatments for Alzheimer's disease: Clinician perspectives

**DOI:** 10.1177/13872877261429859

**Published:** 2026-03-25

**Authors:** Catherine Pennington, Prathima Apurva, Ailin Chen, Alan Duncan, Graham Mackay, Hugh Masters, Katherine Paramore, Tom Russ, Helen Skinner, Martin Zeidler

**Affiliations:** 1 Department of Neurology, NHS Forth Valley, Larbert, UK; 2 Department of Neurology, NHS Lothian, Edinburgh, UK; 3 Centre for Clinical Brain Sciences, University of Edinburgh, Edinburgh, UK; 4 Brain Health Scotland (part of Alzheimer Scotland), Edinburgh, UK; 5 NHS State Hospitals Board for Scotland, Carstairs, Lanark, UK; 6 3035 Older Adult Psychiatry, NHS Dumfries & Galloway, Dumfries, UK; 7 1015 Department of Neurology, NHS Grampian, Aberdeen, UK; 8 3049 Department of Neurology, NHS Fife, Cupar, UK; 9 Department of Old Age Psychiatry, NHS Lothian, Edinburgh, UK

**Keywords:** Alzheimer's disease, brain health, mild cognitive impairment

## Abstract

In recent years there have been exciting developments in the diagnosis and treatment of mild cognitive impairment and dementia due to Alzheimer's disease. Robust biomarkers and potentially disease modifying therapies are now available, with multiple other agents in clinical trials alongside on-going validation studies of blood-based biomarkers. Recent and probable future developments in the diagnosis and care of people with Alzheimer's disease pathology warrants serious re-evaluation of the structure and function of cognitive clinical services. Here we report recommendations from the November 2024 Brain Health Scotland roundtable discussion of opportunities and challenges for modern memory services.

Dementia is one of the greatest societal challenges of the twenty-first century. An estimated 1.4 million are living with dementia in the United Kingdom (UK),^1^ where it the most common cause of death.^2^ A quarter of UK hospital in-patients aged over 65 years have dementia. Alzheimer's disease (AD) is the most common cause,^3^ although mixed neuropathology is frequent^4,5^ as is the phenomena of cognitively healthy older adults having asymptomatic neuropathology, e.g., AD without dementia.^6,7^

Currently available National Health Service (NHS) treatments show only a modest slowing of memory decline in AD dementia.^8^ After decades of waiting, there are now new treatments aiming to modify AD neuropathology.^9,10^ Lecanemab and donanemab are approved by regulators in the United States, European Union, and UK for treatment of mild cognitive impairment (MCI) or dementia due to AD.^11,12^ Neither agent met National Institute for Clinical Excellence (NICE) criteria for clinical use in the UK and are not available to NHS patients at the time of writing.^13–15^ However, it is plausible that these or other novel agents will be approved for NHS usage in the near future,^16^ with multiple challenges to widespread integration to clinical practice.

Key to delivering any novel treatment is setting out benchmarks for clinical efficacy and specifying service structure. In November 2024, Brain Health Scotland held a roundtable discussion forum for clinicians working in cognitive services across Scotland. Here we report clinical recommendations around evaluation and use of novel agents for AD.

Lecanemab and donanemab are monoclonal antibodies targeting regions of the amyloid-β protein.^9,10^ In phase 3 trials, both reduced brain amyloid levels and slowed decline of cognition and quality of life. While they are labelled ‘disease modifying’ based on amyloid clearance, neither stops or reverses cognitive, quality of life or functional decline in people with MCI or dementia due to AD. The crucial unanswered question is whether they are more efficacious than current cholinesterase inhibitors and glutamate receptor antagonists.^17^ The degree of superiority needed to justify usage is heightened by the novel drugs having far greater costs, an intravenous (IV) mode of delivery and significant potential side effects.^18^

Lecanemab is given fortnightly, with emerging evidence that sub-cutaneous delivery may be as effective as IV.^19^ The optimal duration of lecanemab therapy is unclear. A common clinical endpoint in AD clinical trials is the Clinical Dementia Rating – Sum of Boxes (CDR-SB). This uses participant and informant reports to capture functioning in six domains, with higher scores indicating greater impairment. What constitutes a clinically meaningful difference in CDR-SB is a subject of expert debate, a recent review suggested a difference of 1 as representing a meaningful clinical difference.^20^ Open-label extension data for the Lecanemab Clarity AD trial showed a decline of 3.09 on the CDR-SB over 36 months, compared to a decline of 4.04 in an untreated Alzheimer's Disease Neuroimaging Initiative (ADNI) cohort over the same duration.^21,22^ Donanemab is given monthly, with the goal of stopping treatment when amyloid levels fall into the ‘negative’ range on positron emission tomography scanning. In the TRAILBLAZER-ALZ2 study, this was achieved by 76% of participants by week 76.^10^ It is hoped that following amyloid clearance donanemab can be stopped and individuals monitored for amyloid re-accumulation prior to any re-commencement of therapy. Modelling suggests a median amyloid re-accumulation rate of 2.80 CL/year after treatment cessation,^23^ similar to rates seen in untreated adults.^24^ Trial participants who became amyloid negative by week 52 continued to have low amyloid levels at 3 year follow up.^25^ People treated with donanemab showed a 1.2 difference on the CDR-SB at 36 months compared to an untreated ADNI cohort.^26^ Overall, there is good evidence for amyloid reduction with these agents. Currently available data indicates an impact on cognition which may reach clinical significance (around 1 on the CDR-SB), but whether clinical efficacy in the long term is sufficient to justify associated side effects and costs is not yet clear.

Anti-amyloid agents carry a potential risk of serious adverse events. Amyloid-related imaging abnormalities (ARIA) are common, ranging from asymptomatic edema to fatal intracerebral hemorrhage, with most events occurring during the first six months of treatment.^9,10,27^ Most events are asymptomatic, with rates of symptomatic events being less than 3% for lecanemab^28^ and around 5% for donanemab.^29^ Fatal events are rare, and more likely to occur with concurrent anticoagulant use or after thrombolysis for stroke or myocardial infarction. Magnetic resonance imaging (MRI) of the brain is required at baseline and regularly during treatment initiation, plus ad hoc if ARIA occurs. People homozygous for Apolipoprotein ε4 (*APOE4*) have a higher risk of ARIA, hence are specifically excluded from treatment by the UK regulator.^11,12^ Care will be needed to ensure that genetic results are disclosed sensitively to individuals homozygous for *APOE4*, particularly given their increased risk of AD dementia. For all patients the safety versus efficacy of novel agents should be considered alongside the characteristics of existing agents,^17^ and the individual considering treatment should be involved in discussions of risk versus benefit.

Existing UK cognitive services are primarily focused on the diagnosis of dementia, and while there has been a concerted effort to improve rates of early diagnosis, services are highly pressurized. The 2023–2024 National Audit of Memory Assessment Services in England found a mean wait of 5 months from referral to diagnosis, compared to 3 months in 2019.^30^ Only 44% of individuals included in the 2023–2024 audit had any form of brain imaging. NHS England estimates that for adults aged 65 or older living with dementia, only 66.5% have a formal diagnosis recorded.^31^ Delays in diagnosis of neurodegenerative MCI and early dementia limit the ability to influence disease trajectory by risk factor modification, delay the appropriate early usage of current treatments, and restricts the autonomy of affected individuals by denying them early knowledge of their condition while they are still best placed to put in place future planning measures, such as power of attorney. A lack of diagnosis impacts on people with moderate or severe dementia, where there is a major risk of inappropriate treatments or gaps in social care resulting in preventable morbidity and hospital admissions.

Currently there are very few NHS services for people with subjective cognitive decline or MCI. Subjective cognitive decline and MCI are often due to non-neurodegenerative conditions such as medication side effects, sleep problems, or mental health conditions.^32,33^ There is a strong argument for robust evaluation of minor cognitive symptoms, with etiologically based diagnosis and management. This would improve management of treatable causes, alongside the potential to identify early-stage neurodegeneration. A recent survey by Alzheimer's Research UK found that 75% of adults would want to receive information about their future dementia risk, and 88% would seek assessment if they were worried about possible dementia.^34^ Ideally cognitive services should have integrated pathways allowing referral of people with any severity of symptoms, alongside promotion of brain health (Figure 1). All individuals stand to benefit from this approach, from healthy adults looking to reduce their risk of future dementia, through to people with severe dementia requiring expert management of symptoms.

**Figure 1.**
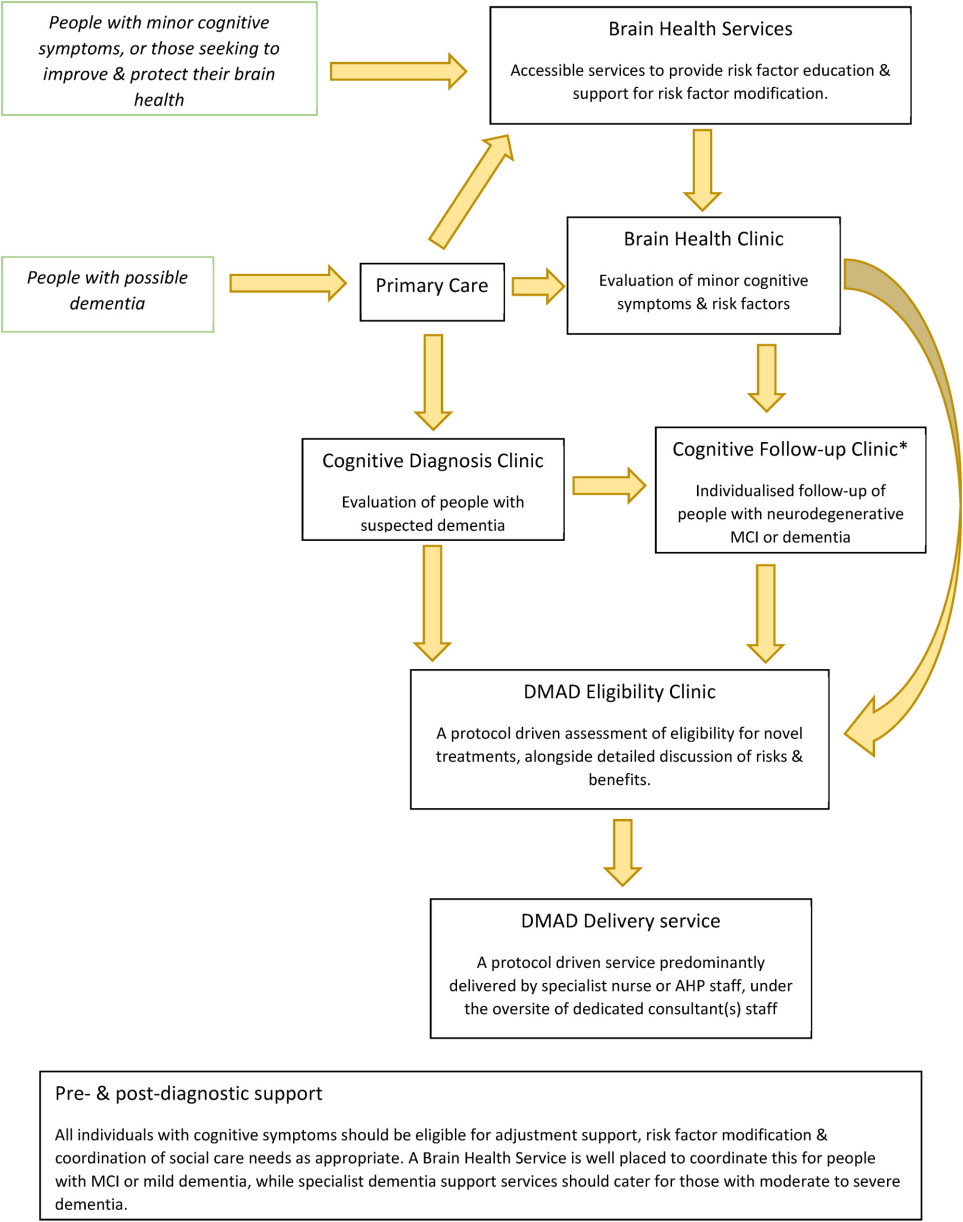
DMAD: disease modifying anti-amyloid drug. * The structure of existing cognitive clinics across the UK is highly variable. Clinical follow-up of patients is heterogenous and depends on resources, clinician behavior and patient age and diagnosis – creating a more standardized level of care across the UK is desirable to reduce healthcare inequalities.

In order to deliver any novel disease modifying therapies a further expansion of services will be needed, with dedicated clinics for eligibility evaluation and service delivery. Additional screening using MRI brain, *APOE* genotyping, and AD biomarkers is likely to be mandatory when assessing suitability for anti-amyloid therapy. NICE included the cost of new clinical services and AD biomarkers for AD pathology in cost evaluations of lecanemab and donanemab.^13–15^ This creates a circular argument against funding memory services—the NHS declines to fund services for people with MCI or the cost of AD biomarkers as new therapies are not approved, while NICE declines to recommend novel treatments due to additional clinical care costs.

AD biomarkers may be a valuable addition to routine cognitive clinic evaluation, regardless of the availability of novel therapies.^35,36^ They could potentially aid in ‘ruling out’ AD pathology in people with MCI and are likely to have far greater utility in that scenario compared to MRI or neuropsychology, at potentially a lower cost. Having greater security in the diagnosis of AD dementia prior to starting cholinesterase inhibitors or glutamate receptor antagonists is highly desirable, particularly for people with unusual cognitive presentations or a young age at symptom onset. Cost savings may occur when blood-based biomarkers for AD become available, as it is very likely that these will displace a certain proportion of neuroimaging tests, particularly the use of nuclear imaging for people with rare presentations.

There is limited data regarding what proportion of individuals with neurodegeneration will be eligible for novel therapies.^27,37,38^ Many people with dementia will be excluded due to the presence of non-Alzheimer's pathology, co-morbidities, anticoagulant use, or *APOE* status. A retrospective review of patients seen in specialist cognitive clinics in London estimated that 14% of patients could be eligible for an anti-amyloid therapy.^38^ Demand will vary substantially across the UK according to patient population and treatment eligibility criteria. Judging the possible uptake by eligible people is also difficult. Early public engagement work has found variable levels of public interest in injectable therapies, ranging from enthusiasm to skepticism about the level of benefit.^39^ Initial numbers accessing any novel treatment will probably be small, but without robust stopping criteria will inevitably grow over time. A potentially attractive approach is using an agent to clear amyloid from the brain, then halting treatment and monitoring amyloid levels. A protocol where short-term treatment is used to push someone into ‘remission’ would be more convenient for those receiving it and lower cost, but only time and further longitudinal data will tell if this is feasible and clinically effective.

We therefore have significant reservations regarding the clinical efficacy of lecanemab and donanemab and the consequences of their significant side effect profile and resource use. Further evaluation of their efficacy for older adults and separately for people with young onset or familial AD is needed. The drug treatment field is rapidly evolving and it is probable that we will soon see other safer and more efficacious AD treatments coming to license, with a variety of modes of action.^16^ We should therefore anticipate the widespread use of novel AD therapies. When planning the delivery of novel agents our priorities should be safety, equitable access and robust data collection alongside concurrent work to improve cognitive services for all patients. Most people with cognitive pathology will not be eligible for novel therapies,^38,40^ and it is vital that their needs are not forgotten.

An integrated pathway should link together Brain Health Clinics, existing memory clinics, and new services focused on novel treatments. Specialist nurses and allied health care professionals already play a key role in many memory services and are likely to be of high value in new services. We advocate for the use of nationally agreed diagnostic, screening and monitoring protocols and close multi-disciplinary team working. By protocolizing diagnostic and eligibility assessments, significant efficiencies can be made, and meaningful data extracted from patient registries. Figure 1 illustrates a potential pathway incorporating services targeting risk reduction, assessment and diagnosis of cognitive disorders; delivery of novel therapies; and post-diagnostic support. Flexibility in service design will be needed to ensure people with rare dementia presentations also receive prompt diagnosis and specialist care. Provision of a dedicated service to assess eligibility for and delivery of anti-amyloid therapies will help alleviate pressure on existing cognitive clinics. Brain health services could play an important role in reducing risk factors for dementia and provide additional capacity for the assessment of people with minor cognitive concerns.^41,42^ There are concerns that services may struggle to meet high demand from people with minor, non-pathological cognitive symptoms. A pilot Brain Health Clinic operated in Aberdeen for 2 years, with a system of self-referral from the public alongside referrals from primary care and public health. The level of public demand was not excessive, with 142 people accessing the service over the 2 year pilot.^43^

An essential part of monitoring treatment response, drug safety and variation in access to services is a compulsory register of people prescribed anti-amyloid therapies. In the United States, the Alzheimer's Association is sponsoring the Alzheimer's Network for Treatment and Diagnostics (ALZ-NET), a voluntary register of individuals receiving novel FDA approved therapies, with the aim of understanding long-term, real world outcomes.^44^ In Japan, post-marketing surveillance is required as part of the lecanemab license, and an academic-run patient register is ongoing. We advocate for a UK wide register of all persons receiving novel anti-amyloid treatments, either as part of an existing initiative such as the International Registry for Alzheimer's Disease and Other Dementias, or using an internationally harmonized dataset.^45^ This would provide robust post-marketing surveillance, additional natural history data, help define the clinical populations most likely to benefit, and aid refinement of imaging and treatment protocols.

Funding for cognitive diagnostic services and research has historically been substantially less than for areas such as cancer,^46,47^ despite the UK and devolved governments having consistently prioritized the timely diagnosis of dementia in recent years. Only 1.4% of healthcare spending on dementia is for diagnosis and treatment.^1^ There are potentially substantial potential savings to health and social care from the earlier diagnosis of dementia, alongside appropriate use of effective novel therapies. It is reasonable to speculate that if effective novel therapies become widely available there will be a significant increase in people seeking medical evaluation of mild cognitive symptoms and early-stage dementia. It is essential that we plan for the implementation of novel therapies for neurodegeneration and use this as an opportunity to improve cognitive services for all patients.

## Key points

- Disease modifying therapies for mild cognitive impairment or dementia due to Alzheimer's disease are likely to reach UK NHS clinics in the near future.
- Significant new investment in cognitive clinics is needed to effectively roll-out novel therapies.
- Most people with neurodegeneration will not be eligible for novel therapies, and their care needs should not be forgotten.
